# Single versus dual elastic nails for closed reduction and antegrade intramedullary nailing of displaced fifth metacarpal neck fractures

**DOI:** 10.1038/s41598-021-81242-3

**Published:** 2021-01-19

**Authors:** Langqing Zeng, Lulu Zeng, Xiaogang Miao, Yunfeng Chen, Weiguo Liang, Yuwen Jiang

**Affiliations:** 1 Department of Orthopaedics, Zhuhai People’s Hospital, Zhuhai Hospital Affiliated With Jinan University, Guangdong, China; 2 Department of Anesthesiology, Zhuhai People’s Hospital, Zhuhai Hospital Affiliated With Jinan University, Guangdong, China; 3 Department of Orthopaedic Surgery, Shanghai Sixth People’s Hospital, Shanghai Jiao Tong University Affiliated Sixth People’s Hospital, Shanghai Jiao Tong University, Shanghai, China; 4 Department of Orthopaedics, Guangzhou Red Cross Hospital, Medical College, Jinan University, Guangzhou, China

**Keywords:** Diseases, Medical research

## Abstract

Closed reduction and internal fixation with antegrade intramedullary nails is a feasible and effective treatment for displaced fifth metacarpal neck fractures (FMNFs). The present study aimed to compare clinical and radiological outcomes in patients with displaced FMNFs after treatment with single or dual antegrade elastic intramedullary nails (AEIMNs). Thirty-three patients were treated with a single 2.0 mm AEIMN and 34 patients were treated with two 1.5 mm AEIMNs. Clinical and radiological outcomes included grip strength, active range of motion (ROM), active flexion and extension of the fifth metacarpophalangeal (MCP) joint, dorsal angulation loss, and metacarpal shortening of the fifth metacarpal at 12 months after treatment. No significant difference was observed between the two groups with respect to grip strength, ROM or flexion of the fifth MCP joint. The average values of dorsal angulation loss, metacarpal shortening, and extension of the fifth MCP joint of the dual nails group were better than those of the single nail group (dorsal angulation loss, 2.79 ± 1.93° vs. 4.05 ± 1.59°, *P* = 0.009; metacarpal shortening, 1.66 ± 0.80 mm vs. 2.12 ± 0.88 mm, *P* = 0.028; extension of the fifth MCP joint, 7.71 ± 4.43° vs. 4.82 ± 4.09°, *P* = 0.012). In conclusion, dual AEIMNs fixation provided better MCP extension and radiological outcomes than single AEIMN fixation.

Fifth metacarpal neck fractures (FMNFs) are considered the most common type of hand fractures; they account for approximately 20% of all fractures in the hand and are more common in males^1,2^. The majority of FMNFs are simple and closed and are generally treated with conservative methods^3^. However, severe palmar displacement and shortening or rotational deformity of the fifth metacarpal fracture may result in a considerable decrease in grip strength and range of motion (ROM), and surgical treatment is recommended for such cases^4,5^. Cadaveric studies suggest that metacarpal head angulations greater than 30° result in dysfunction of small finger motion at the metacarpophalangeal (MCP) joint^6^. Various techniques are available for treating FMNFs, including closed reduction with percutaneous pinning, antegrade or retrograde intramedullary nailing, open reduction and internal fixation with plates and/or screws, and transverse pinning with k-wires^4,5,7–10^. However, there is as yet no consensus on the most ideal technique of fixation.

The goal of surgical treatment is to restore hand function, and not to simply heal the hand as observed in normal radiographs. In general, the surgical technique should be able to minimize soft tissue disruption and allow early motion of the hand^8^. Recently, the use of single or dual antegrade elastic intramedullary nailing (AEIMN) has gained increasing interest because it is relatively simple, causes minimal trauma, is cosmetically acceptable, reduces the risk of soft tissue adhesions, and shows good to excellent clinical outcomes^4,11–14^. Yammine et al.^12^ reported that antegrade intramedullary nailing provided better clinical and radiological outcomes than percutaneous transverse pinning or miniplate fixation in their meta-analysis of treatment procedures for FMNFs. The features of internal fixation, including the number and diameter of the nail, are associated with the stability of fixation and therefore may further affect the secondary displacement and prognosis. However, to the best of our knowledge, no previous studies have compared both single and dual elastic intramedullary nailing techniques in terms of the stability of fixation and clinical and radiological outcomes. In this study, FMNFs were surgically treated with a closed reduction and percutaneous antegrade intramedullary nailing fixation with single or dual elastic nails. The authors evaluated whether the number of nails affected the stability of elastic intramedullary nailing fixation and the clinical and radiological outcomes.

## Materials and methods

This retrospective study was conducted in accordance with the Declaration of Helsinki and was approved by the Ethics Committee of Zhuhai People’s Hospital. Informed consent was obtained from all donors. Between February 2012 to July 2018, 78 patients with isolated displaced FMNFs were treated with closed reduction and percutaneous antegrade elastic intramedullary nailing (Double Medical Technology INC, Xiamen, China).

Inclusion criteria were as follows: patients over 18 years of age who had an isolated FMNF (≤ 14 days), dorsal angulation of the metacarpal neck of ≥ 30°, or presence of rotational deformity of the fifth finger upon flexion. Patients meeting any of the following criteria were excluded: any injuries on tendons, ligaments, vessels, and nerves on the ipsilateral upper limbs; multi-fragmentary fractures, open fractures, or noncooperative patients. Eleven patients were excluded due to incompletion of a 12-month follow-up.

Sixty-seven patients (54 males and 13 females) completed the study. Thirty-three patients were treated with single AEIMN (single nail group). Thirty-four patients were treated with dual AEIMNs (dual nails group). All surgeries were performed by the same two surgical orthopaedic specialists (LQ Zeng and YW Jiang). Surgeries were performed under general anaesthesia in 15 patients and under brachial plexus block in 52 patients. All surgeries were performed under fluoroscopic guidance, and all fractures were closed reduced.

**Figure 1:**
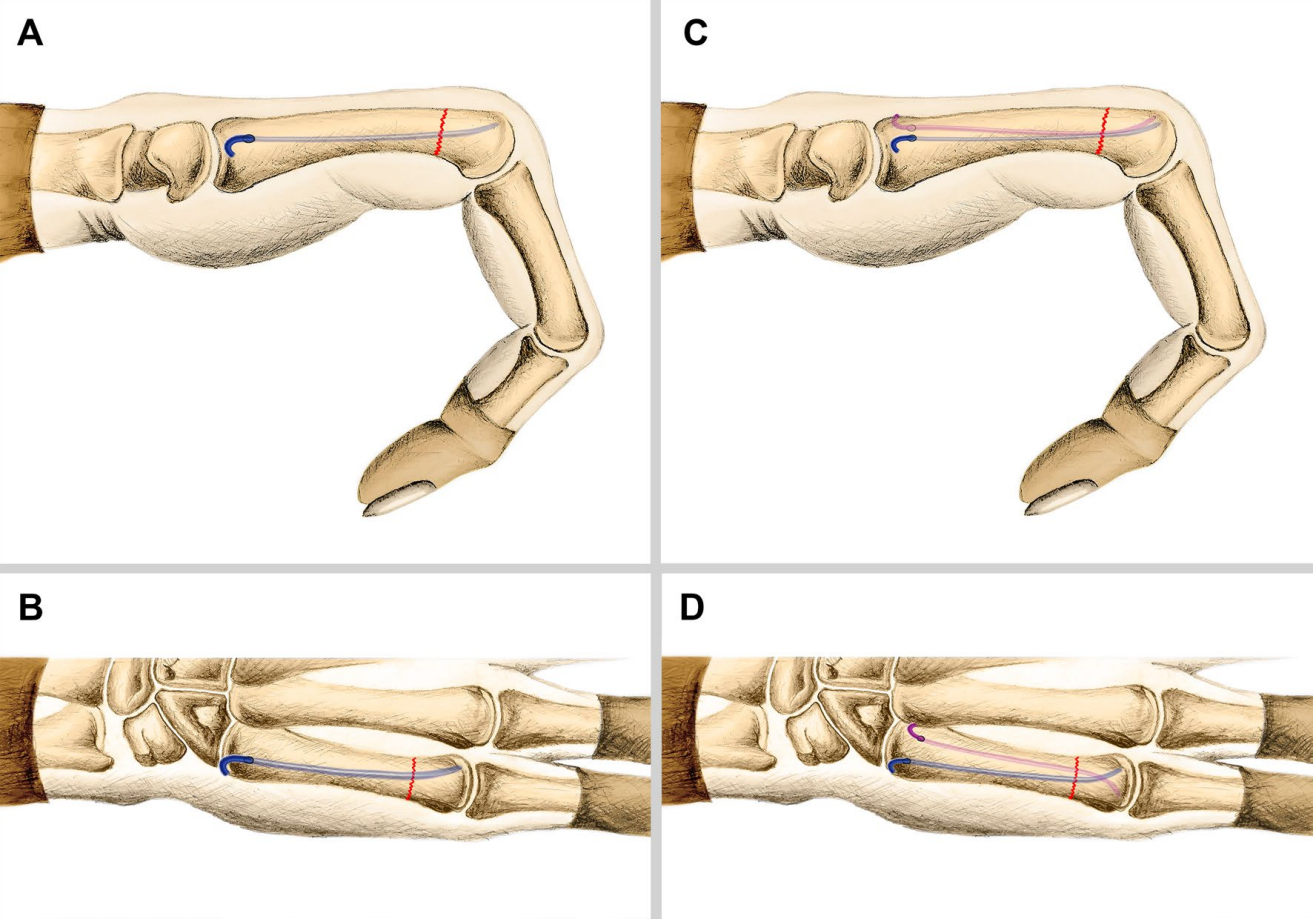
Schematic illustration of an FMNF fixed with single AEIMN on lateral view (**A**) and anteroposterior view (**B**), and fixed with dual AEIMNs on lateral view (**C**) and anteroposterior view (**D**).

**Figure 2:**
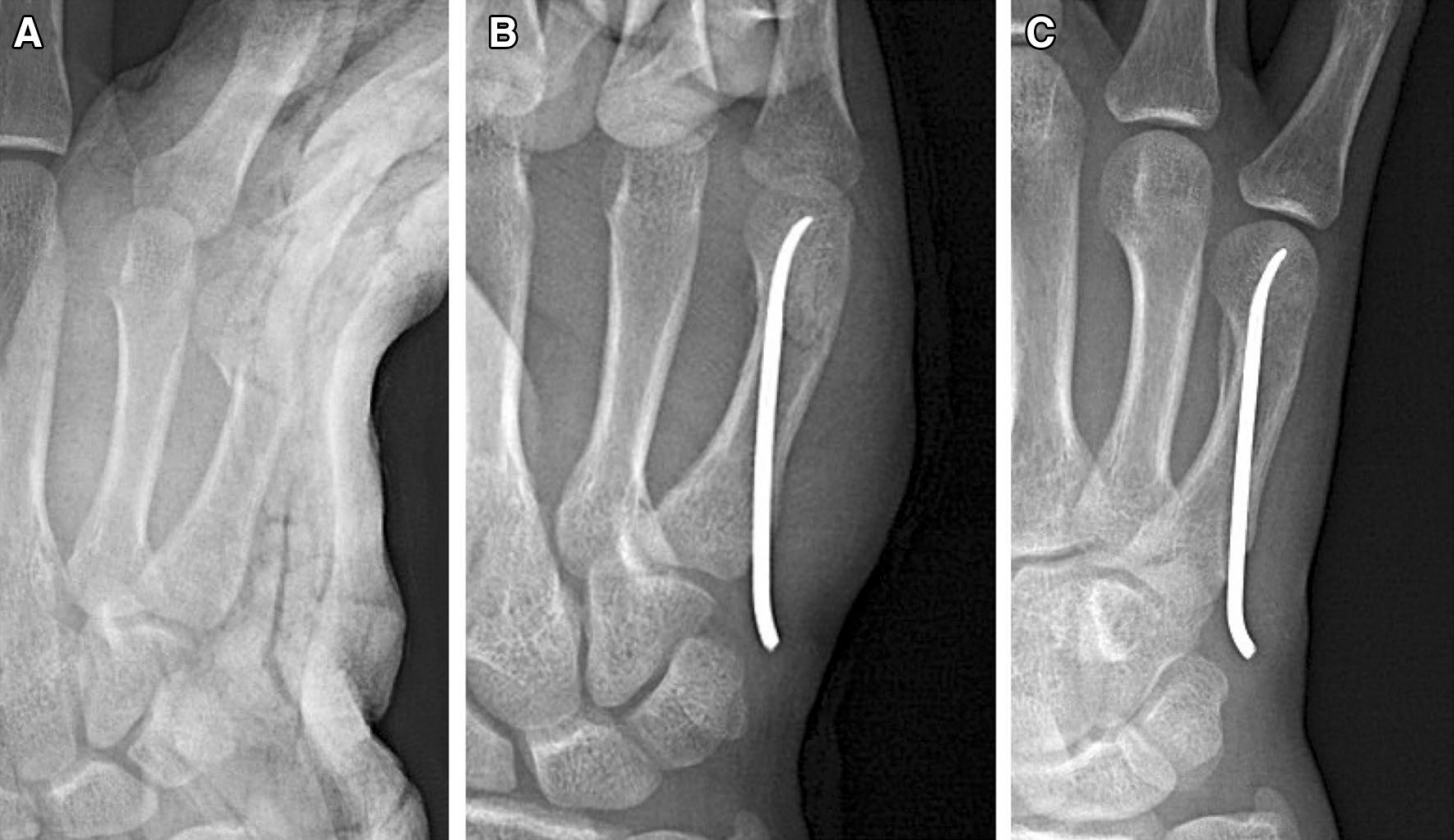
Clinical case of an FMNF treated by single AEIMN. Preoperative oblique view radiograph (**A**). Postoperative radiographs showing a good reduction of the fracture (**B**)**.** Radiograph at 8 weeks after the operation shows union of the fracture (**C**).

For single AEIMN, a small incision was made at the dorsal-ulnar aspect of the metacarpal base. The fifth metacarpal base was approached by a subcutaneous blunt dissection. An awl was used to open the dorsal-ulnar cortex in an oblique manner. An elastic nail of 2.0 mm in diameter was selected, and it was inserted into the medullary cavity and advanced antegrade to the fracture. After the fracture was reduced, the nail was advanced until the nail tip reached the subchondral bone of the metacarpal head, with the tip of the nail directed at the dorsal surface of the metacarpal head. This allows for a 3-point fixation that increases the stability of fixation (Fig. 1A,B). The proximal end of the nail was bent and cut, leaving approximately 8 mm extending out of the fifth metacarpal, and then buried subcutaneously (Fig. 2A–C).

**Figure 3:**
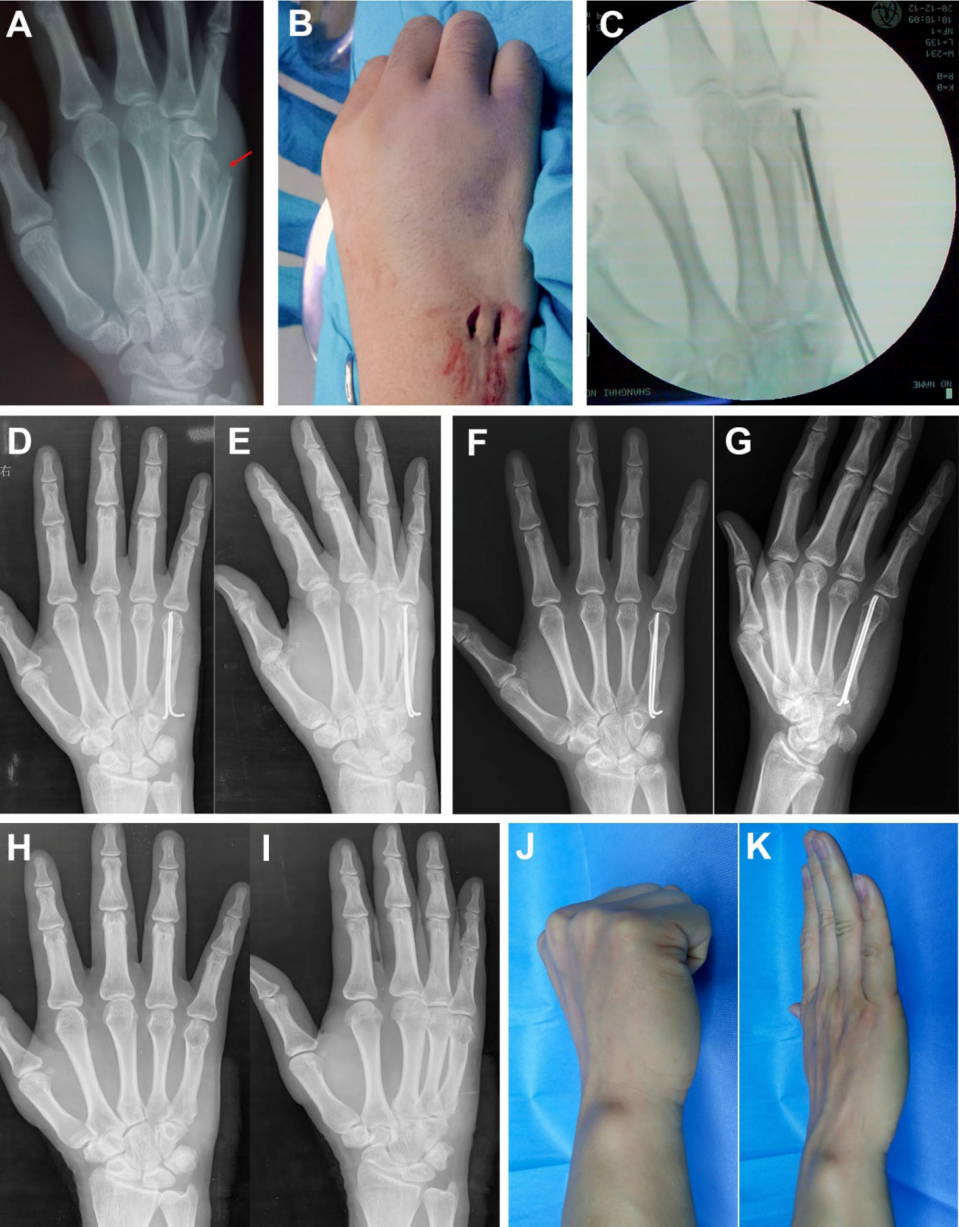
Clinical case of an FMNF treated by dual AEIMNs. Preoperative oblique view radiograph (**A**). Intraoperative photograph showing two incisions in the metacarpal base (**B**)**.** Intraoperative fluoroscopy (**C**). Postoperative radiographs showing good reduction of the fracture on the anteroposterior view (**D**) and oblique view (**E**)**.** Radiograph at 8 weeks after the operation shows union of the fracture (**F, G**). Radiograph at 6 months after removal of the nails (**H, I**). Photographs of the hand in flexion (**J**) and extension (**K**) at 12 months after operation.

For dual AEIMNs, two small incisions were made at the dorsal-ulnar and dorsal-radial aspect of the metacarpal base. The fifth metacarpal base was approached by a subcutaneous blunt dissection, and an awl was used to open the dorsal-ulnar and dorsal-radial cortex in an oblique manner. Two elastic nails of 1.5 mm in diameter were inserted into the medullary cavity and advanced antegrade to the fracture. After the fracture was reduced, the nails were advanced until their tips reached the subchondral bone of the metacarpal head. The surgeon adjusted the direction of the nail s’ tips so that they were directed at the dorsal surface of the metacarpal head (Fig. 1C) to form a cross configuration at the level of the nail tips (Fig. 1D). The proximal ends of the nails were bent and cut, leaving 5 mm extension out of the fifth metacarpal, and then buried subcutaneously (Fig. 3A–K).

Postoperatively, the MCP joints were left free (without using the splint), and gentle passive motion was initiated. After 1–2 weeks, active motion was started. At 4 weeks, strengthening exercises and light daily activities such as writing and computer work were allowed. Activities requiring power grips such as sports and heavy works were allowed after union of the fractures. The patients were followed up at 2, 4, and 6 weeks, and at 3, 6, and 12 months. Radiographic follow-up included an anteroposterior view and a 30° oblique view hand X-ray. Clinical union was defined as the absence of tenderness at the fracture site^15^. In both groups, the elastic nails were removed 3–6 months later under locoregional anaesthesia in the operating room of the outpatient surgery department.

**Figure 4:**
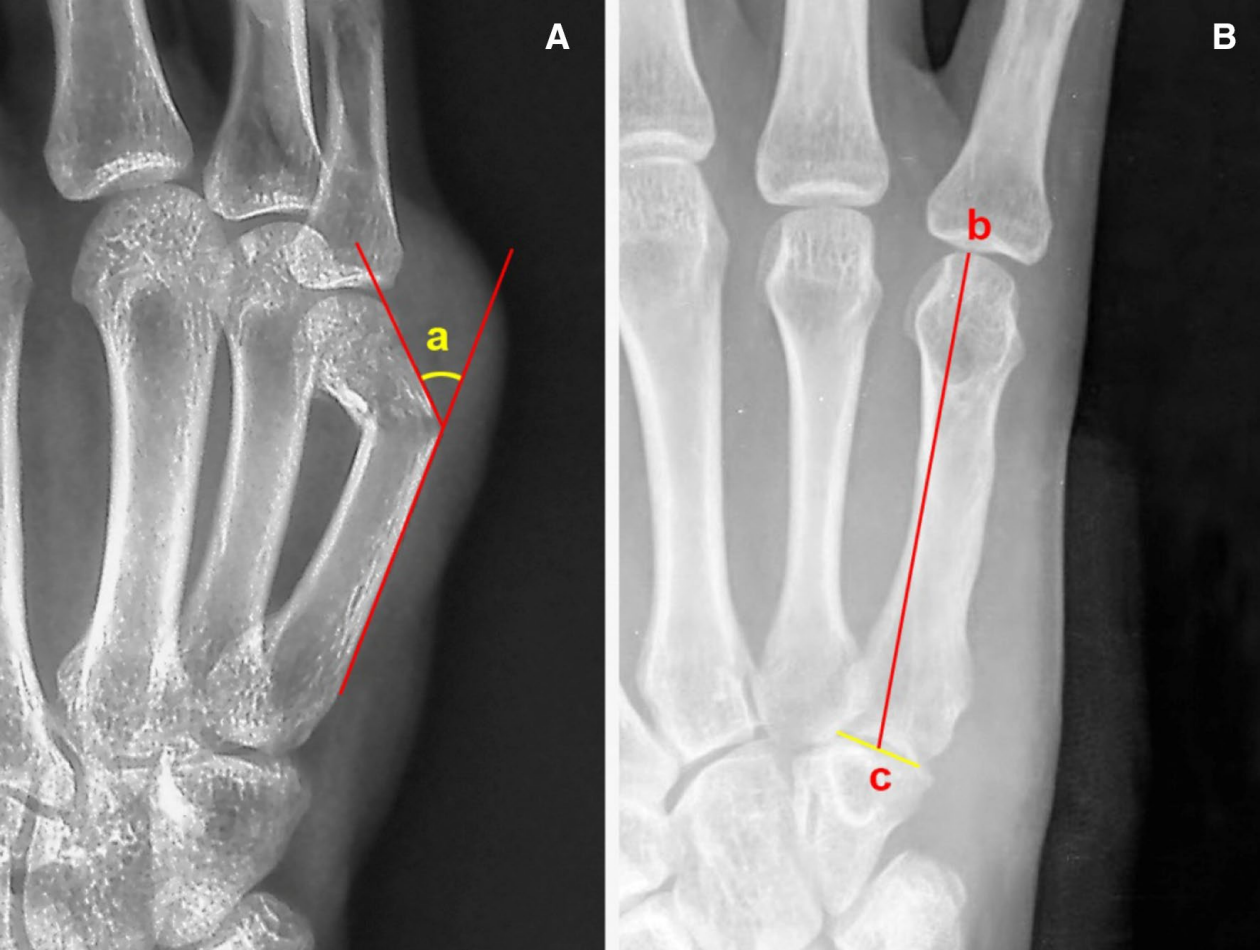
Methods of radiological measurements. Dorsal angulation was defined as an acute angle (**a**) between the line drawn on the dorsal cortex of the fifth metacarpal shaft and the second line on the dorsal cortex of the fifth metacarpal head/neck (**A**). The metacarpal length was defined as the length between the most distal articular surface of the fifth metacarpal head (**b**) and the mid-point (c) of both corners of the fifth metacarpal base (**B**)^16^.

Demographic parameters, including age, gender, injury mechanism, dominant hand, time to treatment, and operative time, were recorded for both groups. An independent assessor blinded to patient details assessed the radiological and clinical outcomes. For radiological assessments, dorsal angulation and metacarpal length were radiologically measured using a picture archiving and communication system. The dorsal angulation was measured on the 30° pronated oblique view in the true lateral hand position (Fig. 4A), and the length of the fifth metacarpal was measured on the anteroposterior view (Fig. 4B)^16^. The secondary dorsal angulation loss and metacarpal shortening of the fifth metacarpal from postoperative radiographs acquired immediately after surgery to 12 months after operation were calculated. Clinical functions of the hand were assessed at 12 months after operation. For clinical assessment, the short version of the Disabilities of the Arm, Shoulder, and Hand questionnaire (Quick-DASH score) was used^15,17^, and the visual analogue scale (VAS) was used to evaluate pain in the hand. Grip strength in percentage (%) compared to the contralateral side was measured using a Jamar’s dynamometer (Asimov Engineering, Los Angeles, CA, USA)^18^. The time to return to work was recorded. The active ROM of the fifth MCP joint was measured by a handheld goniometer, in terms of active flexion and extension of the MCP joint^19^.

Complications were noted at all follow-ups, including loss of reduction, non-union, penetration of the nail tip through the metacarpal head, infection, tendon irritation, tendon adhesion, skin irritation, and injury to the dorsal cutaneous branch of the ulnar nerve. Loss of reduction was defined as the secondary angulation of the metacarpal neck of ≥ 30° in the follow-up.

Descriptive statistics were expressed using mean ± standard deviation for normally distributed variables and median (interquartile range [IQR]) for non-normally distributed variables. Comparisons between groups were analysed by the chi-square test and Fisher’s exact test for categorical outcomes, and Student’s *t* test and nonparametric Wilcoxon rank-sum test for continuous outcomes. *P* < 0.05 was considered statistically significant. SAS 11.0 (SAS Institute Inc., Cary, NC, USA) was used for statistical analysis.

## Results

There were 33 patients with single AEIMN fixation and 34 patients with dual AEIMNs fixation. In our series, 54 patients were males and 13 patients were females. The average age of this series of patients was 31.09 ± 9.94 (18–67 years). The right hand was affected in 43 patients, and the dominant hand was affected in 47 patients. There were no significant differences between the two groups with respect to age, sex, side of injury, dominant hand, time from initial trauma to operation, preoperative dorsal angulation, and mechanism of injury (Table 1). However, the operative time was significantly longer in the dual nails group than in the single nail group (*P* < 0.000) (Table 1).

**Table 1:** Patient demographic data of both groups.

Characteristics	Single nail group (n = 33)	Dual nails group (n = 34)	Statistics	*P*
Age (years)	30.30 ± 8.69	31.85 ± 11.11	0.63	0.528
Sex (male/female)	27/6	27/7	0.062	0.803
Side of injury (right/left)	22/11	21/13	0.175	0.676
Injured dominant hand (n)	23	24	0.006	0937
Time to operation (days)	3.09 ± 1.01	2.88 ± 0.88	− 0.90	0.371
Angulation pre-op (°)	45.87 ± 9.20	45.17 ± 9.18	− 0.31	0.756
Operation time (min)	25.91 ± 4.96	37.27 ± 8.53	6.63	< .000
Injury mechanism (n)			0.232	0.972
Fall	4	3		
Crush	6	7		
Punch	18	19		
Sports	5	5		

No significant difference was observed between the two groups with respect to the dorsal angulation immediately post-operation, the dorsal angulation at 12 months after operation, bone union time, Quick-DASH score, VAS score, MCP joint flexion, ROM of the MCP joint, grip strength, and time to return to work (Table 2). The dorsal angulation loss was significantly greater in the single nail group (4.05 ± 1.59°) than in the dual nails group (2.79 ± 1.93°) (*P* = 0.009). The single nail group showed significantly greater metacarpal shortening (2.12 ± 0.88 mm) than the dual nails group (1.66 ± 0.80 mm) (*P* = 0.028). However, the MCP joint extension was significantly better in the dual nails group (7.71 ± 4.43°) than in the single nail group (4.82 ± 4.09°) at 12 months post-operation (*P* = 0.012) (Table 2).

**Table 2:** Clinical and radiological outcomes of both groups.

Characteristics	Single nail group (n = 33)	Dual nails group (n = 34)	Statistics	*P*
Angulation post-op (°)	10.60 ± 3.63	10.34 ± 3.69	− 0.30	0.766
Angulation post-op. 12 months (°)	14.65 ± 3.53	13.13 ± 3.70	− 1.71	0.091
Angulation loss (°)	4.05 ± 1.59	2.79 ± 1.93	− 2.70	0.009
Metacarpal shortening (mm)	2.12 ± 0.88	1.66 ± 0.80	− 2.25	0.028
Bone union time (weeks)	10.06 ± 2.03	10.35 ± 2.00	0.59	0.555
Quick-DASH score	7.27 ± 5.08	6.62 ± 4.52	− 0.56	0.579
VAS score	0.55 ± 0.79	0.56 ± 0.75	− 0.23	0.820
MCP extension (°)	4.82 ± 4.09	7.71 ± 4.43	2.57	0.012
MCP flexion (°)	90.27 ± 5.56	90.50 ± 7.61	0.14	0.887
ROM of MCP (°)	95.09 ± 6.89	98.21 ± 7.52	1.78	0.079
Grip strength (%/healthy side)	91.46 ± 9.09	91.62 ± 10.43	0.07	0.946
Time return to work (weeks)	8.82 ± 2.38	8.74 ± 2.60	− 0.14	0.892
Complication rate (n%)	7 (21.21%)	6 (17.65%)	0.14	0.712

*DASH* Disabilities of the arm, shoulder, and hand questionnaire; *VAS* visual analogue scale; *MCP* metacarpophalangeal joint; *ROM* range of motion.

There was no loss of reduction, non-union or malunion, infection, or tendon adhesion complications in any patient in this study. The most common complication was skin irritation at the entry point of the nails (six patients), including three patients each in the single and dual nail groups. These six patients were successfully treated by a second operation to remove the nails under locoregional anaesthesia. Migration of the nail tip into the MCP joint was observed in three patients (including two patients in the single nail group and one patient in the dual nails group), but it did not influence the final outcome. Three patients had injury to the dorsal cutaneous branch of the ulnar nerve, including two patients in the single nail group and one patient in the dual nails group. In addition, one patient experienced tendon irritation of the extensor digitorum minimi tendon at the end of the dorsal-radial nail in the dual nails group. This patient was successfully treated by a second operation to remove the nails. No significant difference was noted in the rates of complications between the two groups (*P* = 0.712) (Table 2).

## Discussion

Various surgical modalities have been reported for treating FMNFs, and each modality has its own potential advantages and disadvantages. Percutaneous K-wire fixation offers limited soft tissue disruption, but it could also lead to an increase in superficial infection and irritation of the skin^20^. In addition, K-wires may lead to unstable fracture reduction and require auxiliary immobilization by splint after operation, which delays the ability for early motion. Plate osteosynthesis has the advantage of immediate stabilization of the fracture^21^. However, this technique has the disadvantage of excessive soft tissue damage, which may lead to complications such as tendon adhesion, severe tendon irritation, and joint stiffness^22,23^. Retrograde intramedullary nailing fixation with K-wires, or elastic nails have been reported for the treatment of FMNFs, but these techniques cause tendon irritation and MCP stiffness^24^. The use of a retrograde headless intramedullary screw has shown good outcomes^25,26^. The main advantage of this technique is faster recovery to perform daily living and work-related activities, with no serious complications^27^. However, this technique damages articular cartilage. Ten Berg et al.^28^ reported that the articular surface injury caused by the screws in the metacarpal head has a relatively low relevance (4% for the 2.4 mm screw and 5% of the total joint surface for the 3.0 mm screw). To date, there have been no reports of mid-term osteoarthritic degeneration at the metacarpal head following the use of this technique.

Antegrade intramedullary osteosynthesis with K-wires or elastic nails offers limited soft tissue stripping, does not affect the joint capsule, reduces the risk of soft tissue adhesion, has excellent functional and cosmetic results, and lowers severe complication rate^4,12,29^. Thus, these techniques have become a commonly used method for the fixation of FMNFs. The intramedullary nailing fixation acted on a three-point intramedullary fixation, thereby providing adequate stability. Although the biomechanical data showed that intramedullary nailing fixation was less stable than plate osteosynthesis, the former is significantly stronger in monocyclic loading than crossed K-wire osteosynthesis^30^. Heo et al.^16^ and Foucher^31^ suggested that intramedullary nailing fixation can provide adequate stability to allow early mobilization. It is important that early postoperative mobilization is allowed to reduce the risk of stiffness. Previously, Kim et al. reported that the antegrade intramedullary pinning group showed better recovery in the ROM of the fifth MCP joint, grip strength, and DASH score than the retrograde group at 3 months after surgery^32^. Winter et al.^6^ and Sletten et al.^22^ also reported that this minimally invasive intramedullary nailing fixation had a better functional recovery than transverse fixation^33,34^. A meta-analysis conducted by Yammine and Harvey showed that antegrade intramedullary pinning provided better grip strength, fifth digit ROM, lower pain scores, and fewer complications than percutaneous transverse pinning or miniplate fixation for the treatment of FMNFs^12^.

In the present study, we showed that the functional outcomes of both single and dual nail groups were satisfactory at 12 months after surgery and the complication rate in both groups was similar and acceptable. These results were found to be similar to those reported in the literature when using single AEIMN or dual AEIMNs^4,11,13,35^. On the other hand, we found that the fixation with dual elastic intramedullary nails could reduce the secondary displacement (both dorsal angulation loss and metacarpal shortening) and improve the MCP extension as compared to that with single elastic intramedullary nailing fixation. However, the dual nails group needed more operative time than the single nail group, because the intraoperative manipulation of dual AEIMNs is relatively complicated. Calder et al. treated FMNFs with a blunt 1.6 mm K-wire, and they reported an average volar angulation of 3.7° and an average metacarpal shortening of 3.8 mm^36^. Boonyasirikool C and Niempoog S in their anatomical study showed that the average metaphyseal widths of metacarpal bone were between 11.42 and 16.42 mm and the a medullary canal widths were between 3.05 and 6.74 mm^37^. Therefore, intramedullary fixation with two 1.5 mm elastic nails is a practicable technique for the majority of adult FMNFs. On the basis of our experience, elastic nails are easier to manipulate during the operation than K-wires, because the nails are more flexible and the distal tips of the nails have a natural curve; thus, they do not require bending. In addition, fixation with two 1.5 mm elastic intramedullary nails may provide more biomaterial stability than fixation with a single 2.0 mm elastic intramedullary nail. Hence, for patients requiring an early return to activity, fixation with two 1.5 mm elastic intramedullary nails may be preferred. Malik et al.^38^ stated that the normal angulation of the fifth metacarpal head to the neck is 15 degrees. In our opinion, an adequate reduction of the fractures should be achieved with dorsal angulation less than 15 degrees and without rotational deformity. The entry holes of the elastic nails were made in the dorsal cortex of the fifth metacarpal base (Fig. 1). When the fixation is being completed, the surgeon needs to adjust the direction of the nail’s tip toward the dorsal surface of the metacarpal head (Fig. 1). Thus, a three-point fixation is created with two dorsal contact points at the base and head of the fifth metacarpal and one palmar contact point at the fifth metacarpal’s shaft (Fig. 1). This configuration may provide more biomaterial stability because it is in the opposite direction to the natural dorsal convexity of the fifth metacarpal^15^. Furthermore, the surgeon needs to adjust the direction of the nails’ tips to form a cross configuration at the level of the tips for dual nail fixation (Fig. 1D). This configuration may provide more biomaterial stability, because the nails’ tips point in opposite directions. No severe complications occurred in any patient. Both single and dual AEIMN fixations had complications of skin irritation, protrusion of the nail tip into the MCP joint, and injury to the dorsal cutaneous branch of the ulnar nerve. We believe that the tail of the nails should be bent, cut to an appropriate length, and buried subcutaneously. The bent tail might prevent forward migration of the nail tip and avoid being covered with bone. The direction of the nail’s tail may need to be adjusted to avoid its contact with tendons and to reduce its prominence after skin closure. These manipulations might reduce the risk of skin and tendon irritation. On the other hand, instead of direct puncture, we could make dissection incisions and spread subcutaneous tissue bluntly to avoid iatrogenic injury to the dorsal cutaneous branch of the ulnar nerve^4^.

This study has several limitations. First, although the radiological outcomes were blinded assessment, there may have been a measurement error in radiological parameters. Second, the quality of radiographs might have influenced the measurement results, because it was difficult to acquire the oblique radiograph under certain conditions. Finally, because the number of patients was relatively small and the follow-up time was only 12 months, the results may not be reproducible in other centres with different surgical indications. A larger, long-term, multi-centre, prospective study is therefore required to appropriately address these issues.

## Conclusion

The present study showed that both single and dual AEIMN fixations are safe and effective treatment options for FMNFs. Better MCP extension and less dorsal angulation loss and metacarpal shortening are advantages of dual AEIMN fixation over single AEIMN fixation. Hence, for patients requiring an early return to activity, fixation with two 1.5 mm elastic intramedullary nails may be preferred. Early motion after AEIMN fixation should be performed carefully because complications related to articular perforation or reduction loss may occur.
